# Chemosensitivity Predicted by BluePrint 80-Gene Functional Subtype and MammaPrint in the Prospective Neoadjuvant Breast Registry Symphony Trial (NBRST)

**DOI:** 10.1245/s10434-014-3908-y

**Published:** 2014-08-07

**Authors:** Pat Whitworth, Lisette Stork-Sloots, Femke A. de Snoo, Paul Richards, Michael Rotkis, Jennifer Beatty, Angela Mislowsky, James V. Pellicane, Bichlien Nguyen, Laura Lee, Charles Nash, Mark Gittleman, Stephanie Akbari, Peter D. Beitsch

**Affiliations:** 1 Department of Surgery, Nashville Breast Center, Nashville, TN USA; 2 Department of Medical Affairs, Agendia NV, Amsterdam, The Netherlands; 3 Department of Medical Oncology, Blue Ridge Cancer Care, Roanoke, VA USA; 4 Department of Surgery, Northern Indiana Cancer Research Consortium, South Bend, IN USA; 5 Department of Surgery, The Breast Place, Charleston, SC USA; 6 Department of surgery, Coastal Carolina Breast Center, Murrells Inlet, SC USA; 7 Department of surgery, Virginia Breast Center, Bon Secours Cancer Institute, Richmond, VA USA; 8 Department of Medicine, Todd Cancer Institute, Long Beach Memorial Medical Center, Long Beach, CA USA; 9 Department of Surgery, Comprehensive Cancer Center, Palm Springs, CA USA; 10 Department of Medical Oncology, Northeast Georgia Medical Center, Gainesville, GA USA; 11 Breast Care Specialists, Allentown, PA USA; 12 Department of Surgery, Virginia Hospital Center, Arlington, VA USA; 13 Department of Surgery, Dallas Surgical Group, Dallas, TX USA

## Abstract

**Purpose:**

The purpose of the NBRST study is to compare a multigene classifier to conventional immunohistochemistry (IHC)/fluorescence in situ hybridization (FISH) subtyping to predict chemosensitivity as defined by pathological complete response (pCR) or endocrine sensitivity as defined by partial response.

**Methods:**

The study includes women with histologically proven breast cancer, who will receive neoadjuvant chemotherapy (NCT) or neoadjuvant endocrine therapy. BluePrint in combination with MammaPrint classifies patients into four molecular subgroups: Luminal A, Luminal B, HER2, and Basal.

**Results:**

A total of 426 patients had definitive surgery. Thirty-seven of 211 (18 %) IHC/FISH hormone receptor (HR)+/HER2− patients were reclassified by Blueprint as Basal (*n* = 35) or HER2 (*n* = 2). Fifty-three of 123 (43 %) IHC/FISH HER2+ patients were reclassified as Luminal (*n* = 36) or Basal (*n* = 17). Four of 92 (4 %) IHC/FISH triple-negative (TN) patients were reclassified as Luminal (*n* = 2) or HER2 (*n* = 2). NCT pCR rates were 2 % in Luminal A and 7 % Luminal B patients versus 10 % pCR in IHC/FISH HR+/HER2− patients. The NCT pCR rate was 53 % in BluePrint HER2 patients. This is significantly superior (*p* = 0.047) to the pCR rate in IHC/FISH HER2+ patients (38 %). The pCR rate of 36 of 75 IHC/FISH HER2+/HR+ patients reclassified as BPLuminal is 3 %. NCT pCR for BluePrint Basal patients was 49 of 140 (35 %), comparable to the 34 of 92 pCR rate (37 %) in IHC/FISH TN patients.

**Conclusions:**

BluePrint molecular subtyping reclassifies 22 % (94/426) of tumors, reassigning more responsive patients to the HER2 and Basal categories while reassigning less responsive patients to the Luminal category. These findings suggest that compared with IHC/FISH, BluePrint more accurately identifies patients likely to respond (or not respond) to NCT.

Classification by molecular subtype has been recommended as a guide for the selection of therapy for patients with breast cancer. However, at present, the methodology for molecular subtyping is not standardized and the methodology and interpretation of results vary between different laboratories. Subtype is being assigned using conventional immunohistochemistry (IHC) and fluorescence in situ hybridization (FISH) (“conventional subtype”) or molecularly using gene expression profiling.

Neoadjuvant trials allow for rapid assessment of treatment sensitivity, and pathological complete response (pCR) has been proposed as a surrogate endpoint for longer-term outcome. One recently available molecular profile is BluePrint. The profile determines the mRNA levels of 80 genes that discriminate between three breast cancer subtypes based on functional molecular pathways: Luminal, HER2, and Basal.^1^ A further stratification of the Luminal group into types A and B is important to identify the risk of metastasis and has been related to tumor grade and/or proliferation (Ki-67 fraction or mitosis).^2^ However, risk stratification by multigene assays, such as MammaPrint, is superior for making this distinction, whereby the MammaPrint low-risk patients are identified as Luminal A and MammaPrint high risk corresponds to Luminal B.

In a retrospective analysis, the molecular stratification of patients with BluePrint and MammaPrint was used to correlate the response to neoadjuvant chemotherapy and long-term outcomes in patients with early-stage or locally advanced breast cancer. Patients (*n* = 435) had been enrolled in four, independent, neoadjuvant chemotherapy clinical trials (I-SPY 1 trial, two trials at the University of Texas M.D. Anderson Cancer Center, and a trial from the City of Hope National Medical Center). The pCR rate differed substantially in the different molecular subgroups: 6 % in the Luminal A; 11 % in Luminal B; 48 % in the HER2; and 37 % in the Basal groups. Luminal A (MammaPrint Low Risk) patients had a good prognosis with excellent survival and seemed not to benefit from chemotherapy. A marked benefit in response and DMFS to neoadjuvant treatment in patients subtyped as HER2 and Basal was observed.^3^

The objective of the current prospective NBRST study is to compare chemosensitivity as defined by pCR or endocrine sensitivity as defined by partial response (PR) for patients classified with the 80-gene BluePrint functional subtype profile to conventional IHC/FISH subtyping.

## Patients and Methods

### Patients

Patients with histologically proven breast cancer, who had started or were scheduled to start neoadjuvant chemotherapy therapy or neoadjuvant hormone therapy after successful MammaPrint/BluePrint assay, were enrolled in the prospective NBRST registry trial between June 2011 and October 2013 from 40 institutes in the United States. The protocol was approved by institutional review boards at all participating institutions. All patients provided written, informed consent for participation in the study. Excluded from the study were patients who had an excisional biopsy or axillary dissection, confirmed distant metastatic disease, any prior chemotherapy, radiotherapy, or endocrine therapy for the treatment of breast cancer and any serious uncontrolled, intercurrent infections, or other serious uncontrolled concomitant disease. Treatment was at the discretion of the physician adhering to NCCN approved or other peer-reviewed, established regimens.

### Molecular and Clinical Subtyping

Fresh (*n* = 120) or formalin fixed paraffin embedded tumor samples (*n* = 306) were obtained from core needle biopsies. Microarray analysis (RNA labelling, microarray hybridization, and scanning) for obtaining the 80-gene BluePrint subtype and 70-gene MammaPrint profiles was performed at the centralized Agendia Laboratory blinded for clinical and pathological data. RNA was cohybridized with a standard reference to the custom-designed diagnostic chip, each containing oligonucleotide probes for the profiles in triplicate or more.

BluePrint stratifies into three distinct molecular subgroups: Luminal (BPLuminal), HER2 (BPHER2), and Basal (BPBasal). MammaPrint substratifies BPLuminal into Luminal A (BPLuminalA for MammaPrint Low Risk) and Luminal B (BPLuminalB for MammaPrint High Risk). Hormone receptor (HR) status (ER and PR) and HER2 status were determined locally on pretreatment core biopsies. Both ER and PR status were determined by IHC and were considered positive if there was ≥1 % positive staining. HER2 status was determined by IHC and/or FISH assays locally. HER2 status was regarded as positive if there was 3+ staining and/or FISH positivity.

### Statistical Analysis

In the ongoing NBRST registry, for neoadjuvant chemotherapy patients the primary endpoint is pCR, which is defined as the absence of invasive carcinoma in both the breast and axilla at microscopic examination of the resection specimen, regardless of the presence of carcinoma in situ. PR is defined as ≥30 % reduction in the tumor area. PR is a secondary endpoint for neoadjuvant chemotherapy patients and the primary endpoint for neoadjuvant endocrine therapy patients. Rates of pCR were calculated for each BluePrint/MammaPrint molecular subtype and compared with pCR rates for subgroups classified by IHC/FISH. The response rates are presented as a proportion of all patients treated with NCT. Comparison of response rates is conducted using a two-tailed *z*-test for two population proportions. The null hypothesis is that there is no difference between these two population proportions. Hence, a *p* value less than the significance level of 0.05 means that the null hypothesis cannot be accepted; proportions are different. All calculations were performed with SPSS statistical package version 16.0 (SPSS, Chicago, IL).

## Results

A total of 426 patients (age range 22–82), T1-4 N0-3, underwent surgical resection and had pCR information available. Clinical characteristics are shown in Table 1. Of the 426 patients, 59 (14 %) were classified as BPLuminalA, 153 (36 %) patients were classified as BPLuminalB, 74 patients (17 %) were classified as BPHER2, and 140 patients (33 %) were classified as BPBasal. According to pathological assessment, 211 patients (50 %) were IHC/FISH HR+/HER2−, 123 (29 %) were IHC/FISH HER2+ (of whom 75 were HR+ and 48 were HR−), and 92 (22 %) patients were IHC/FISH triple-negative (TN). Most patients had T2 or T3 tumors (85 %) and clinically or pathologically confirmed axillary lymph node involvement (56 %) at time of diagnosis; 93 % had tumors of intermediate or high histologic grade; 86 % of patients were classified as high risk by MammaPrint.

**Table 1:** Clinical characteristics for patients as classified according to BluePrint and MammaPrint

Characteristic	Luminal A	Luminal B	HER2	Basal	Total
	(*n* = 59)	(*n* = 153)	(*n* = 74)	(*n* = 140)	(*N* = 426)
Median age (range), years	57 (33–78)	54 (22–80)	52 (23–73)	51 (28–79)	52 (22-80)
T stage					
1	3	13	5	17	38
2	33	92	38	88	251
3	20	38	24	31	113
4	3	10	6	4	23
Missing	0	0	1	0	1
N stage					
0	30	48	22	65	165
1–3	24	95	48	70	237
Missing	5	10	4	5	24
Grade					
1	10	7	2	1	20
2	43	73	29	20	165
3	5	66	43	119	233
Missing	1	7	0	0	8
MammaPrint					
Low risk	59	0	2	0	61
High risk	0	153	72	140	365

### IHC Versus BluePrint/MammaPrint-Based Classification

We evaluated the distribution of patients within the conventional IHC/FISH subclassification and as reclassified by BluePrint molecular subtype as illustrated in Table 2. In total, 22 % (94/426) of patients were reclassified into a different BluePrint/MammaPrint molecular subgroup compared with conventional (IHC/FISH) subtyping; 37 of 211 (18 %) IHC/FISH luminal (HR+/HER2−) patients were not BPLuminal (35 BPBasal and 2 BPHER2). Fifty-three of 123 (43 %) IHC/FISH HER2+ patients were not BPHER2 (36 BPLuminal and 17 BPBasal). Four of 92 (4 %) IHC/FISH TN patients were not BPBasal (2 BPLuminal and 2 BPHER2). The BPLuminal patients were further stratified with MammaPrint into BPLuminalA (*n* = 59) and BPLuminalB (*n* = 153).

**Table 2:** Conventional (IHC/FISH) subtype versus BluePrint/MammaPrint molecular subtype

IHC/FISH	BluePrint/MammaPrint				Total
	Luminal A	Luminal B	HER2	Basal	
HR+/HER2−	51	123^a^	2	35^b^	211
HER2+ (HR+)	8	28	33	6	75
HER2+ (HR−)	0	0	37	11	48
Triple negative	0	2	2	88^c^	92
Total	59	153	74	140	426

^a^5 HER2 IHC/FISH equivocal

^b^1 HER2 IHC/FISH equivocal

^c^5 HER2 IHC/FISH equivocal

### Neoadjuvant Treatment

A total of 280 (66 %) patients received NCT without trastuzumab of whom the majority (92 %) received a regimen containing anthracyclines and taxanes; 123 (29 %) patients received NCT with trastuzumab (2 patients received trastuzumab and pertuzumab), 65 % received docetaxel/carboplatin/trastuzumab (TCH), and 35 % doxorubicin/cyclophosphamide followed by docetaxel/trastuzumab (AC-TH). Three (<1 %) patients received NCT and NET, 20 (5 %) NET (Tables 3, 4).

**Table 3:** Neoadjuvant treatment received by BluePrint/MammaPrint molecular subtyping

Treatment	BluePrint/MammaPrint				Total
	Luminal A	Luminal B	HER2	Basal	
NCT	37	116	4	123	280
NCT/trastuzumab	7^a^	29^b^	70	17^c^	123
NCT/NET	0	3	0	0	3
NET	15	5	0	0	20
Total	59	153	74	140	426

^a^7 IHC/FISH HER2+ patients

^b^25 IHC/FISH HER2+ patients, 2 IHC/FISH HER2 equivocal patients, and 2 with a positive mRNA HER2 read out

^c^16 IHC/FISH HER2+ patients and 1 IHC/FISH HER2 equivocal patient

**Table 4:** Neoadjuvant treatment received by IHC/FISH conventionally classified subtypes

	IHC/FISH			Total
	Luminal (HR+/HER2−)	HER2+	Triple negative	
NCT	183	6	91	280
NCT/trastuzumab	5^a^	117	1^b^	123
NCT/NET	3	0	0	3
NET	20	0	0	20
Total	211	123	92	426

^a^2 IHC/FISH equivocal patients, 1 BluePrint HER2, and 2 with a positive mRNA HER2 read out

^b^1 IHC/FISH equivocal patient

### Response Rates to Neoadjuvant Chemotherapy Treatment

**Fig. 1:**
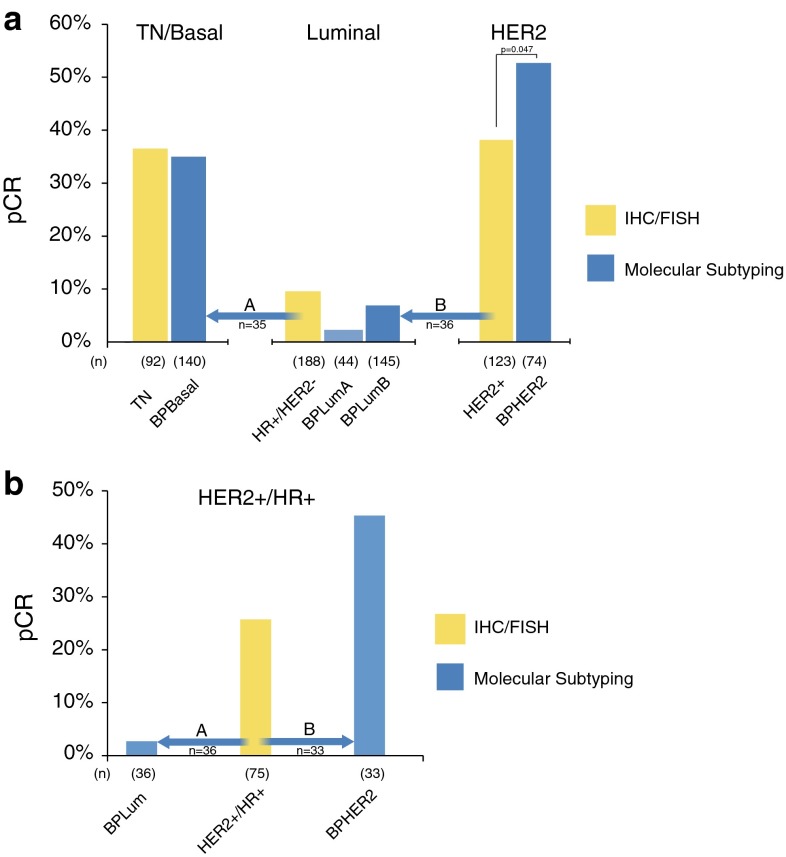
**a** pCR rates and major subtype re-assignments for patients classified by BluePrint/MammaPrint molecular subtyping compared with IHC/FISH assessed subgroups. The analysis includes only patients treated with NCT (*n* = 403). The two major subtype reassignments were (A) conventional luminal (HR+/HER2−) patients, 35 of 188 (19 %) patients reclassified by BluePrint as Basal (*arrow A*) and (*B*) conventional HER2+ patients, 36 of 123 (29 %) reclassified by BluePrint as Luminal (*arrow B*). **b** pCR rates and major subtype reassignments for conventional HER2+/HR+ (“triple positive”) patients (95 % treated with NCT/trastuzumab). Thirty-six of 75 (48 %) of conventional HER2+/HR+ patients were reclassified by BluePrint as Luminal—with only 1 pCR (3 %) to NCT (*arrow A*). Thirty-three of 75 (44 %) of conventional HER2+/HR+ patients were classified by BluePrint as HER2, with a pCR rate to NCT of 45 % (*arrow B*). Six conventional HER2+/HR+ patients were reassigned to BPBasal (not shown)

The overall pCR rate to NCT was 99 of 403 (25 %). The pCR rates of the IHC/FISH subclasses and BluePrint/MammaPrint molecular subclasses are shown in Fig. 1a. Note that 23 patients were not treated with NCT, and these patients are not included in the response to neoadjuvant chemotherapy analyses. The pCR rate in BPLuminal patients who received NCT was 11 of 189 (6 %: 2 % BPLuminalA and 7 % BPLuminalB) versus 18 of 188 (10 %) in IHC/FISH HR+/HER2− patients. The pCR rate in BPHER2 patients was 39/74 (53 %) and was significantly superior to the 47/123 (38 %) in IHC/FISH HER2+ patients (*p* = 0.047). Of the 140 BPBasal patients, including the 35 reclassified from the IHC/FISH HR+/HER2− and 17 from the IHC/FISH HER2+ categories, all received NCT; 49 (35 %) had a pCR, similar to the pCR rate 34/92 (37 %) seen in the patients originally designated TN by IHC/FISH.

### Response Rates for Reclassified Patients

The pCR rate in IHC/FISH HR+/HER2− (conventional luminal) patients was 10 %. However, 35 of 188 (19 %) of conventional luminal patients were classified by BluePrint as Basal (Fig. 1a, arrow A). The pCR rate in these patients was 26 % (Table 5).

**Table 5:** BluePrint/MammaPrint subtype pCR rates within the different conventionally classified subtypes (IHC/FISH) treated with NCT (*n* = 403)

IHC/FISH	HR+/HER2−		HER2+/HR+		HER2+/HR−		TN		Total	
	*n*	pCR (%)	*n*	pCR (%)	*n*	pCR (%)	*n*	pCR (%)	*n*	pCR (%)
Total	188	10	75	27	48	56	92	37	403	25
Molecular subtypes										
Luminal A	36	3	8	0	–	–	–	–	44	2
Luminal B	115	7	28	4	–	–	2	50	145	7
HER2	2	0	33	45	37	65	2	0	74	53
Basal	35	26	6	67	11	27	88	38	140	35

Of conventional HER2+ patients, 36 of 123 (29 %) were reclassified by BluePrint as Luminal (Fig. 1a, arrow B). All 36 came from the subset of IHC/FISH HER2+ patients who were HR+ (“triple positive”). When IHC/FISH HER2+ patients were subdivided into those who were hormone receptor-positive (HER2+/HR+) versus negative (HER2+/HR−), the pCR rate in HER2+/HR+ was inferior to that for HER2+/HR−: 20/75 (27 %) versus 27/48 (56 %; *p* = 0.001). Of the 75 HER2+/HR+ (“triple positive”) patients, the 36 who were reclassified by BluePrint as Luminal had only 1 pCR (3 %) to NCT (32 received NCT/trastuzumab) (Fig. 1b, arrow A). This pCR rate is significantly lower than the pCR rate of 45 % in the 33 HER2+/HR+ patients (44 %) who are BPHER2 and received NCT/trastuzumab (*p* < 0.000; Fig. 1b, arrow B). The pCR rate in IHC/FISH HER2+/HR+ reclassified as BluePrint Luminal was 0 % in Luminal A and 4 % in Luminal B (Table 5). Of six IHC/FISH HER2+/HR+ patients reclassified as BPBasal, four had a complete response (all were treated with NCT and trastuzumab).

### Response Rates to Endocrine Treatment

Twenty of 426 (5 %) patients received NET. All patients were IHC/FISH HR+/HER2− and the PR rate was 65 %. Fifteen of 20 (75 %) patients were BluePrint Luminal A and 12 (80 %) patients had a PR. Only one of the five BluePrint Luminal B patients had a PR to NET. Three IHC/FISH HR+/HER2− and BluePrint Luminal B patients received NET and NCT and two had a PR.

## Discussion

Molecular subtype has been suggested as a superior classification to determine treatment strategy for breast cancer patients. Neoadjuvant chemosensitivity and endocrine sensitivity rates are increasingly accepted as surrogates for efficacy, especially if substantial impact can be demonstrated. In this present study when using BluePrint and Mammaprint for defining molecular subtypes, 22 % (94/426) of breast cancer patients are classified in a different subgroup compared with conventional assessment. Treatment was at the discretion of the physician adhering to NCCN-approved or other established, peer-reviewed regimens and is mostly in line with conventional assessment. This reclassification of patients leads to an improved distribution of response rates in the different subgroups of patients: a lower pCR rate for BPLuminal patients compared with IHC/FISH-defined conventional luminal patients, with more responsive patients reassigned to the HER2 and Basal categories.

BluePrint/MammaPrint subtypes have previously been compared to quality-controlled, centrally assessed IHC/FISH subtypes in the first 621 patients of the MINDACT trial.^4^ This analysis showed that 58 % of IHC/FISH HER2+ patients were classified as BluePrint HER2 which is almost identical to the 57 % in the present analysis. This indicates that 42–43 % of conventional HER2+ patients are classified differently by BluePrint molecular subtyping. Conventionally classified TN patients using central IHC/FISH pathology were Basal by BluePrint in 98 % of cases, which is similar to the 96 % in this study. BluePrint almost always reconfirms the basal phenotype of TN patients. As for conventional luminal patients (central pathology determined HR+/HER2−) 96 % also were classified as BluePrint Luminal, 14 % lower in the current study (82 %). The latter higher discordance rate between conventionally classified luminal and BluePrint Luminal patients might be related to the variability in IHC ER and/or PR assessment at local institutions. Accurate test performance is crucial, yet there is evidence of wide variability in test performance and inaccurate results (falsely negative or falsely positive) of up to 20 %.^5^

The observed difference in clinically assessed subgroups of early stage breast cancer patients compared with molecular sub classification of patients has been reported also by others, for instance for subtyping with the intrinsic molecular signature^6^ and the PAM50 signature.^7^,^8^ And even though some of the discordance could potentially be ascribed to technical issues such as test performance and the fact that assessments from different tumour areas are being compared, the discordance also seems to indicate a ‘true’ difference in assigning patients by these 2 types of assessments. Molecular classification is designed such that it captures the true biologic profile regulated by ER/PR/HER and it measures these pathways by measuring a larger number of related genes. The BluePrint 80-gene classifier identifies “functional” molecular subtype based on intact molecular pathways associated with concordant mRNA and protein expression (1).

### Clinical Impact

The two largest groups of reassigned patients with potential clinical implications are those conventional HER2+ patients, who are not classified as HER2 by BluePrint, and the conventional luminal (HR+/HER2−) patients who are reclassified by BluePrint to Basal. The BluePrint HER2 group of patients show a significantly higher pCR rate than that for patients classified as HER2+ by IHC/FISH, with less responsive patients reassigned to the BluePrint Luminal category. All of these reassigned patients come from the HR+ subset of conventional HER2+ patients.

Several studies have suggested that pathologic complete response was not particularly prognostic for ER+, HER2+ breast cancers, suggesting the possibility that a subset of HER2+, ER+ breast cancers are driven primarily by ER, and biologically behave more like HER2−, ER+ breast cancers. Identification of this subset of HER2+ breast cancers is essential to avoid overtreatment of patients with small HER2+, ER+ breast cancers, who may be optimally treated with endocrine therapy alone, or in combination with a HER2-directed agent, thereby avoiding the use of chemotherapy.^9^ In our study, 75 patients are IHC/FISH HER2+/HR+ of whom 36 (48 %) are BluePrint Luminal with a significantly lower pCR rate (3 %) to NCT/trastuzumab versus the 33 IHC/FISH HER2+/HR+ patients (44 %) who are BluePrint HER2 (pCR = 45 %). Therefore with BluePrint functional subtype IHC/FISH HER2+/HR+ patients are subdivided into BPLuminal with a poor response to NCT/trastuzumab and BPHER2 with a good response to NCT/trastuzumab.

An additional 17 (14 %) of 123 conventional HER2 patients were reclassified BPBasal. This finding is likely to become more important as biological subsets within the TN/Basal subtype are delineated.

Many of the conventional luminal patients were reclassified as Basal by BluePrint, enlarging the Basal category while the pCR rate was maintained. This group of conventional luminal patients are reported to have low expression levels of ER and PR and also has been identified by other methods of molecular classification 4,10. For this group of patients conventionally identified as endocrine responsive who are reclassified to the Basal subgroup, it makes sense to consider neoadjuvant chemotherapy.

We have adhered to the recently more stringent definition of pCR: ypT0/is ypN0 as suggested by Cortazar et al. 11. As suggested in this large pooled data analysis, our results can be used to compare response rates as a measure of outcome on a patient level. The overall pCR rate in our study (25 %) is comparable to the overall pCR rate in the Cortazar pooled analyses (22 %). IHC/FISH HR+/HER2− had a pCR rate of 8 % (grade 1 and 2) and 16 % (grade 3) in the pooled analyses, which is similar to the IHC/FISH HR+/HER2− pCR rate of 10 % in our study. Response rates for IHC/FISH HER2+/HR+ (31 vs. 27 %), HER+/HR− (50 vs. 56 %), and TN (34 vs. 37 %) also were comparable. In the pooled analyses of Cortazar et al. 11. The correlation between pCR rate and long-term outcome was strongest for HER2+/HR− patients and TN patients. A limitation of our study is that long-term outcome data are not yet available.

In a retrospective pooled analysis of MammaPrint and BluePrint in patients enrolled in four neoadjuvant chemotherapy trials pCR rate correlated with DMFS in BluePrint HER2 and Basal patients and not in BluePrint Luminal patients.^3^ The current study confirms the unique identification of a group of patients classified as Luminal A using Molecular Subtyping with MammaPrint and BluePrint who have an extremely low pCR rate (2 %) and who have previously been shown to have excellent survival.

These findings confirm the more accurate identification of molecular subgroups for treatment decision by the 80-gene BluePrint functional subtype classifier, which therefore may serve as a better guide for neoadjuvant treatment than standard, local IHC/FISH assay. Approximately one in five conventional “luminal” patients are reclassified as BPBasal and approximately half of conventional HER2+ HR+ patients are reclassified as BPLuminal.
